# Are midwives in the Netherlands satisfied with their jobs? A systematic examination of satisfaction levels among hospital and primary-care midwives in the Netherlands

**DOI:** 10.1186/s12913-019-4454-x

**Published:** 2019-11-13

**Authors:** Doug Cronie, Hilde Perdok, Corine Verhoeven, Suze Jans, Marieke Hermus, Raymond de Vries, Marlies Rijnders

**Affiliations:** 1 OLVG (west) Hospital, Amsterdam, The Netherlands; 2 University of Maastricht, Maastricht, Netherlands; 3 Department of Midwifery Science, AVAG, Amsterdam Public Health research institute, Amsterdam UMC, Vrije Universiteit Amsterdam, Amsterdam, Netherlands; 4 Department of Obstetrics and Gynecology Catharina Hospital, Eindhoven, The Netherlands; 5 Department of Obstetrics and Gynecology Maxima Medical Centre, Veldhoven, The Netherlands; 6 Departmnt of Child Health, TNO*, Leiden, the Netherlands; 7 Royal Dutch Midwives Organization (KNOV), Utrecht, The Netherlands; 8 Faculty of Midwifery Education & Studies, Zuyd University, Maastricht, Netherlands; 9 CAPHRI, School for Public Health and Primary Care, Maastricht University, Maastricht, Netherlands; 10 Center for Bioethics and Social Sciences in Medicine, University of Michigan Medical School, Ann Arbor, MI USA

**Keywords:** Job satisfaction, Survey, Hospital midwife, Primary-care midwife, Integrated-care

## Abstract

**Background:**

Job satisfaction is generally considered to be an important element of work quality and workplace relations. Little is known about levels of job satisfaction among hospital and primary-care midwives in the Netherlands. Proposed changes to the maternity care system in the Netherlands should consider how the working conditions of midwives affect their job satisfaction.

**Aim:**

We aimed to measure and compare job satisfaction among hospital and primary-care midwives in the Netherlands.

**Methods:**

Online survey of all practising midwives in the Netherlands using a validated measure of job satisfaction (the Leiden Quality of Work Questionnaire) to analyze the attitudes of hospital and primary-care midwives about their work. Descriptive and inferential statistics were used to assess differences between the two groups.

**Results:**

Approximately one in six of all practising midwives in the Netherlands responded to our survey (hospital midwives *n* = 103, primary-care midwives *n* = 405). All midwives in our survey were satisfied with their work (*n* = 508). However, significant differences emerged between hospital and primary-care midwives in terms of what was most important to them in relation to their job satisfaction. For hospital midwives, the most significant domains were: working hours per week, workplace agreements, and total years of experience. For primary-care midwives, social support at work, work demands, job autonomy, and the influence of work on their private life were most significant.

**Conclusion:**

Although midwives were generally satisfied, differences emerged in the key predictors of job satisfaction between hospital and primary-care midwives. These differences could be of importance when planning workforce needs and should be taken into consideration by policymakers in the Netherlands and elsewhere when planning new models of care.

**Electronic supplementary material:**

The online version of this article (10.1186/s12913-019-4454-x) contains supplementary material, which is available to authorized users.

## Background

In the Netherlands, maternity care has been traditionally organized around primary-care (where midwives are the lead-professional). The role, remit and autonomy of primary-care midwives to provide low-risk pregnancy-related care is protected by law [1]. Approximately 28% of all midwives in the Netherlands are registered to practise as hospital midwives of which, 100% are employed. The remaining 72% of midwives are registered as primary-care midwives. Of these, 45% are self employed practice owners (practices are further divided into 16% solo practice, 24% duo practice and 60% are part of a group practice). Of the group practices, 40% have 3 or 4 primary-care midwives and 60% consist of group practices with 5 or more primary-care midwives. Of the remaining primary-care midwives, 17% are self-employed agency midwives working only in primary-care, 6% are employed by a midwifery practice (but are not practice owners) and 3% are employed by health or birth centres [2].

Being in employment as part of a large (hospital) organisation means that hospital midwives are part of a different organisational structure and are subject to a different organisational culture than midwives in primary-care. This is can be seen throughout the Dutch midwifery model of care, perhaps most obviously relating to the position of the midwife within the multi-disciplinary team; primary-care midwives are autonomous practitioners, recognised as the lead-carer for women with low-risk, physiological pregnancies. Their status is confirmed within the multi-disciplinary team, whereas, for the hospital midwife this is often not the case. Hospital midwives are frequently the only obstetric professional (in the context of the Dutch maternity care system, the phrase ‘obstetric-professional’ is understood to be limited to: an obstetrician, an obstetrician in training or a hospital midwife) at any one time on shift and commonly look after more than one women in labour at a time. Women birthing in hospitals usually have an increased risk, or known pathology. Hospital midwives usually work fixed periods, typically 8 or 12 h shifts with minimal or no on-call. Whereas primary-care midwives (particularly those in solo, or duo practices routinely work longer hours with greater on-call commitments (24 or even 48 h on-call during weekends are not uncommon). Primary-care midwives, who are paid a per-client fee by insurance companies, will have a caseload of upwards of 100 women per midwife, per year (the recommended maximum norm is 105 women, per midwife, per year [3]. For a fuller explanation of the Dutch maternity care system see: Amelink-Verburg & Buitendijk [4].

In recent years, there has been an increase in the number of midwives working in secondary (hospital) care [2].

Under the present system of care pregnant women who are healthy, as defined by a list of indications [5], are cared for by midwives in the primary care sector. If during pregnancy, a woman develops a condition considered to be “medium or high risk”, she will be transferred to hospital-based care under the care of an obstetrician; in practice however, maternity care in the hospital setting is commonly provided by a hospital-based midwife [6]. This means that care by the primary-care midwife stops at the point of referral to secondary care. This delineation of care has traditionally meant that low risk pregnancy is managed by primary-care midwives and those with increased risk are managed by secondary (hospital) care. Although back and forth referral between primary and secondary care does occur, formal agreements relating to integrated care – where this strict delineation between the primary and secondary care sectors is blurred – are rare. In practice, there is little-to-no continuity of care when a woman is referred from primary to secondary care.

Following the publication of a report detailing perinatal mortality statistics across European nations, where it appeared that perinatal mortality in the Netherlands was higher than that of comparable countries, a strategic review of maternity services was commissioned [7]. The review panel was of the opinion that strict delineation of care may affect collaboration between maternity service providers. As a direct result, changes to the midwifery service provision in the Netherlands are being considered, with the emphasis shifting toward a so-called model of integrated care [8] where the separation between primary and secondary care will become less rigid.

New integrated models of care are likely to result in changes in the working patterns of midwives and may affect their job satisfaction. It is important, therefore, to benchmark current levels of job satisfaction and to more fully explore the drivers of job satisfaction among midwives prior to changes in the system of care.

The concept of job satisfaction is generally accepted to consist of many aspects such as: job autonomy, potential for development and financial reward, working relationships, work demands, social support at work, workplace agreements, organization, and the influence of work on private life [9].

Furthermore, job satisfaction is also seen as an important element of work quality and workplace relations [10]. Studies show a correlation between job satisfaction and efficiency [11], productivity [12], wellbeing [11], as well as the working atmosphere [13]. In the nursing, midwifery and medical professions, ways of working which lead to increased job satisfaction have been shown to improve patient safety, reduce costs, and increase the quality of patient/client experience [14–17].

Existing, small-scale (qualitative) studies considering the views of midwives show that direct client contact, continuity of care, positive support, teamwork, and the ability to work independently and autonomously lead to higher levels of satisfaction [18, 19]. In this study, we have measured Dutch midwives’ job satisfaction. Furthermore, we compare satisfaction levels between hospital and primary-care midwives and examine the factors associated with job satisfaction. Our study of job satisfaction offers a comprehensive view of the opinion of midwives under the current system of care and identifies the drivers of job satisfaction, important knowledge for policymakers, in the Netherlands and elsewhere, who are considering changes in the provision of maternity care.

## Methods

### Design

This is a quantitative study using a cross sectional, self-administered, online questionnaire.

### Sample

The study population is all practising midwives in the Netherlands (*N* = 3150). Our sample includes 103 hospital midwives and 405 primary-care midwives.

### Data collection

Invitations were sent by email to 452 (out of a total of 532^2^) midwifery practices that included an e-mail address on their website. During the first week of March 2015, these practices received a personal email containing information about the study and a link to the survey. In this email, we asked that an link to the survey was forwarded to all midwives in the practice inviting their participation in the study The survey was open to all and accessible via Survey Monkey from February 2015 until April 2015.

An email was also sent to the head-of-department in all hospitals in the Netherlands with maternity-care facilities (*n* = 91) asking them to forward the email invitation to participate to all colleagues (including hospital midwives).

In addition to this direct approach, we also used snowball sampling. The Royal Dutch Organization of Midwives (KNOV) placed a notification on their website, asking all members to participate in this study. Each midwife was asked to distribute the recruiting email among other colleagues after completion of the survey. A first reminder email was sent to all hospitals and midwifery practices after 4 weeks and further reminders were placed on the KNOV website and forums, such as the hospital midwives’ group within the KNOV. Following this, no further reminders were sent.

There was no restriction on the number of participants per hospital or practice. Data were stored electronically in an encrypted database.

### Measures

The Leiden quality of work questionnaire [9] (LQWLQ), was used to measure job satisfaction. The formulation of the questions was adjusted for maternity-care professionals in consultation with the author of the questionnaire (see Additional files 1 and 2 for Dutch and English versions of questionnaire). In total, the questionnaire consists of ten domains, each containing several factor-statements. Nine of the domains explore various elements *related* to job satisfaction and one domain is specifically focused on job satisfaction. This ‘job satisfaction scale’ (α = 0.805) consists of the following six factor-statements: “If I had to choose, I’d choose this job again”/“I would like to change my job”/“I’m satisfied with my job”/“I would recommend this job to a friend”/“When I applied, this was the job that I wanted”/“I often have to do work that I’d rather not do”.

Respondents were asked to answer each statement using a four-point scale (ranging from 1: totally disagree to 4: totally agree). The domain “job satisfaction” was defined as the mean of the six statements. A higher mean score indicated a higher level of job satisfaction. Negatively formulated questions were reversed for analysis.

In addition, we collected information about respondents’ demographic characteristics such as gender, age, years of experience in profession and current organisation, as well as employment status and average working hours per week.

We defined two categories of midwife in the Netherlands. They were: ‘hospital midwife’ (midwife was employed in a hospital in the secondary or tertiary-care sector) or ‘primary-care midwife’ (midwife was self-employed or employed by a primary care midwifery practice). In addition, respondents were offered the option of ‘other’ if their occupation did not fit these categories.

The questionnaire was piloted prior to the survey by five midwives from the primary and secondary care sectors. This resulted in the simplification of two questions and the deletion of one that had been included twice in the draft questionnaire.

### Statistical methods

We compared hospital midwives with primary-care midwives. A mean score for each of the domains for each group was calculated. We used t-tests and linear regression to assess the differences in job satisfaction between the two groups. We then used a bivariate analysis (Pearson’s correlation coefficient) to examine the strength of the within-group relationship between each of the domains and mean job satisfaction. Lastly, we used multiple linear regression to examine the effect of several independent variables (personnel and organisation, work demands and tasks, social support at work, working relationships, workplace agreements and referrals, autonomy, potential for development, financial reward, and the influence of work on private life) on job satisfaction. A *p*-value of 0.05 or lower was considered statistically significant.

IBM SPSS version 22.0 (SPSS, Inc., Chicago, IL, USA) was used for data analysis. Descriptive statistics, chi-square, bivariate and multi-variable regression analyses were performed and normality of the outcome measures was examined. In calculating means, we excluded those with more than one missing item on that scale.

### Ethical considerations

This study forms part of larger study [20] that was submitted to the Ethics Committee of the Vrij Universiteit Medical Centre, Amsterdam (Medical Ethics Committee VuMc, email: metc@vumc.nl, submission reference number: 2014/030. Ethics committee approval was deemed unnecessary, according to national regulations [21]. Therefore, the Committee waived the need for ethics approval.

Consent to participate was implied by completion and return of the questionnaire. The authors have no conflicts of interest.

## Results

Given the method of recruitment (i.e. invitations to midwifery practices and hospital departmental heads and via the KNOV notification on their noticeboards), we are unable to determine the precise response rate. However, given that there are 3150 practising midwives in the Netherlands at the time of the study and that we had 508 midwife respondents, we estimate that our sample includes almost 17% of all practicing midwives in the country.

Fifty-eight of the 566 questionnaires we received were excluded because they were incomplete. Analysis of the incomplete responses showed that these data were missing completely at random (MCAR). No further analysis was performed on these MCAR data. Data were found to be sufficiently normally distributed to allow parametric testing. None of the midwife respondents used ‘other’ to describe their practice area. Hospital midwives make up 28% of all midwives in the Netherlands; our sample included 11% of hospital midwives and 18% of primary-care midwives.

The majority of respondents were female (> 98%). In addition, although the numbers were small, men were over represented in the hospital-midwife category: the national average is < 2% and in our survey it was 4%.

The distribution of postcodes showed that all 12 provinces in the Netherlands were represented. We received responses from 97% of all maternity-hospitals (*n* = 89) in the Netherlands.

Table 1 shows the demographics of our sample population. The mean age of all respondents was 40 years. Hospital midwives were slightly older than primary-care midwives (42 yrs. vs. 38 yrs., *p* = < 0.001). Hospital midwives were significantly more likely to work fewer hours (*n* = 29, 95% CI 27–30, *p* = < 0.001) compared to primary-care midwives (*n* = 44, 95% Confidence Interval [CI] 42–45, *p* = < 0.001). In addition, the range of working hours per week was significantly smaller for hospital midwives than primary-care midwives (28 vs. 142 respectively, *p* = < 0.001). Hospital midwives had slightly more experience compared to primary-care midwives (16 yrs., 95% CI 14.2–17.8 vs. 13 yrs., 95% CI 11.9–13.74 respectively, *p* = < 0.002). All hospital midwives were salaried employees (100%). The majority (77%) of primary-care midwives were self-employed.

**Table 1:** Demographic characteristics of our sample

	Hospital midwives^a^ n (%)	Primary-care midwives^b^ n (%)	*P* value
total	103 (100)	405 (100)	
Gender			
Male	4 (4)	4 (1)	0.05
Female	99 (96)	401 (99)	
Mean Age (±SD)	42 (9.8)	38 (10.5)	0.001
Employed	103 (100)	77 (19)	0.001
Self employed	0 (0)	313 (77)	0.001
Total years of work experience Mean (SD)	16 (9.0)	13 (8.9)	0.002
Years of experience at current work place Mean (SD)	9.3 (7.0)	9.7 (7.9)	0.59
Working hours per week (SD)	29 (5.6)	44 (14.9)	0.001

^a^Total number of hospital midwives registered in the Netherlands: *N* = 919

^b^Total number of primary care midwives registered in the Netherlands: *N* = 2231

Table 2 shows the difference in means between hospital and primary-care midwives on the individual statements from the domain of ‘job satisfaction’. Overall, both groups of midwives were either satisfied or very satisfied with their jobs (mean satisfaction level was: satisfied for combined groups: 3.13, 95% CI 2.9–3.16).

**Table 2:** Mean scores^a^ [standard deviation] for hospital and primary-care midwives for questions in ‘job satisfaction’ domain

Individual factor statements for ‘job-satisfaction’ domain	Overall mean for both professional groups mean [SD]	Hospital midwives^b^ mean [SD]	Primary care midwives^c^ mean [SD]	*P* value
If I had to choose again, I’d choose this job	3.12 [0.68]	3.15 [0.64]	3.02 [0.74]	0.54
I would like to change jobs	3.18 [0.71]	3.02 [0.72]	3.03 [0.76]	0.28
I’m satisfied with my job	3.21 [0.59]	3.06 [0.59]	3.13 [0.63]	0.31
I would recommend a friend to take this job	2.87 [0.67]	2.90 [0.64]	2.62 [0.72]	0.001
When I applied this is the job that I wanted	3.36 [0.55]	3.25 [0.52]	3.27 [0.59]	0.70
I often have to do work that I would rather not do	3.03 [0.57]	3.04 [0.53]	2.86 [0.64]	0.11
Domain score per profession (mean) SD	3.13 [0.47]	3.07 [0.48]	3.02 [0.48]	0.45

^a^Each question had a maximum possible score of 4

^b^*n* = 103

^c^*n* = 405

Mean job satisfaction between the two groups of midwives did not differ significantly. Hospital midwives showed a mean satisfaction of 3.07 (95% CI 2.97–3.17) compared to 3.02 (95% CI 2.98–3.08) for primary-care midwives.

When the mean scores for the factor-statements of the domain ‘job satisfaction’ were analyzed individually, no differences emerged with the exception of hospital midwives, who were significantly more likely to recommend their job to a friend (*p* = 0.001).

Table 3 shows the mean scores for hospital and primary-care midwives for all other domains. For both groups of midwives, the three domains with the highest overall scores were ‘personnel and organization’, ‘social support at work’ and ‘potential for development’. There were differences between groups in these scores with primary-care midwives generally scoring higher. These differences were statistically significant for the following domains: ‘personnel and organization’ (*p* = 0.001), ‘social support at work’ (*p* = 0.001), ‘work demands and tasks’ (*p* = 0.001), ‘autonomy’ (*p* = 0.001) and ‘influence of work on private life’ (*p* = 0.001).

**Table 3:** Mean scores [standard deviations] for hospital and primary-care midwives for other domains

Domain	Overall mean both groups (SD)	Mean score hospital midwives^a^ (SD)	Mean score primary-care midwives^b^ (SD)	*P* value
Personnel & organization	3.17 [0.37]	3.00 [0.30]	3.21 [0.38]	0.001
Work demands & tasks	2.60 [0.25]	2.49 [0.24]	2.63 [0.25]	0.001
Social support at work	3.03 [0.30]	2.93 [0.22]	3.06 [0.31]	0.001
Working relationships	2.74 [0.28]	2.78 [0.23]	2.74 [0.29]	0.190
Workplace agreements & referrals	2.19 [0.31]	2.20 [0.30]	2.19 [0.32]	0.759
Autonomy	2.70 [0.33]	2.52 [0.28]	2.74 [0.33]	0.001
Potential for development	2.98 [0.39]	2.91 [0.40]	2.99 [0.39]	0.080
Financial reward	2.58 [0.60]	2.70 [0.56]	2.55 [0.61]	0.024
Influence of work on private life	2.60 [0.63]	2.13 [0.47]	2.72 [0.60]	0.001

^a^*n* = 103

^b^*n* = 405

Table 4 shows the differences between hospital and primary-care midwives in the within-group correlation of the means of all the other domains to the mean of ‘job satisfaction’ using Pearson’s correlation coefficient.

**Table 4:** Within-group correlation (P_r_) between job satisfaction and each of the other domains for hospital and primary-care midwives

Domain	Correlation coefficient hospital-care midwives^a^ (*p* value)	Correlation coefficient primary-care midwives^b^ (*p* value)
Personnel & organisation	0.404 (0.001)	0.297 (0.001)
Work demands & tasks	0.314 (0.002)	0.380 (0.001)
Social support at work	0.526 (0.001)	0.410 (0.001)
Working relationships	0.356 (0.001)	0.264 (0.001)
Workplace agreements & referrals	0.448 (0.001)	0.251 (0.001)
Autonomy	0.522 (0.001)	0.370 (0.001)
Potential for development	0.514 (0.001)	0.499 (0.001)
Financial reward	0.316 (0.002)	0.271 (0.001)
Influence of work on private life	−0.025 (0.811)	0.256 (0.001)

^a^*n* = 93

^b^*n* = 382

Overall, the correlations for each of the domains with job satisfaction were higher for hospital midwives. For hospital midwives, strong correlations were observed in three domains: ‘social support at work’ (*r* = 0.526), ‘autonomy’ (*r* = 0.522), and ‘potential for development’ (*r* = 0.514). For primary-care midwives, the strongest correlations were in the domains: ‘potential for development’ (*r* = 0.514), ‘social support at work’ (*r* = 0.410), and ‘work demands and tasks’ (*r* = 0.380).

For each group, we looked more closely at the factor-statements *within* each domain that contributed most strongly to the relation between that domain and job satisfaction. For hospital midwives, the domain with the strongest correlation was ‘social support at work’. This domain has eight factor-statements. Of these, the factor-statement “I experience the other partners in the care-chain more like colleagues than competitors” (data not shown) revealed the strongest correlation to job satisfaction. For primary-care midwives the strongest correlation to job satisfaction was ‘potential for development’. This domain has five factor-statements. The factor-statement with the strongest correlation to job satisfaction was “In my work, I can develop sufficiently”.

Table 5 shows the results of a linear regression analysis that used demographic characteristics (age, years of work experience, years at current workplace and working hours-per week) plus the nine domains associated with job satisfaction (personnel and organization, work demands and tasks, social support at work, working relationships, workplace agreements and referrals, autonomy, potential for development, financial reward and the influence of work on private life) to predict job satisfaction for each group.

**Table 5:** Regression model predicting job satisfaction for hospital and primary-care midwives

Model variables	Hospital midwives^a^		Primary-care midwives^b^	
	Beta	95%C.I.lower/upper	Beta	95%C.I.lower/upper
Age	−0.089	− 0.017/0.008	0.031	− 0.006/0.009
Total years of experience in profession	−0.259	− 0.026/− 0.001	−0.157	− 0.019/0.002
Total years of experience in current job	0.146	0.003/0.022	0.050	−0.005/0.12
Working Hours per week	0.170	0.002/0.027	0.016	−0.002/0.003
Personnel & Organization	0.042	−0.227/0.361	0.068	−0.029/0.203
Work demands & tasks	0.171	--0.020/0.627	0.186	0.116/0.376
Social support at work	0.185	−0.028/0.851	0.199	0.168/0.451
Work relationships	0.001	−0.359/0.364	0.034	−0.094/0.208
Workplace agreements & referrals	0.220	0.077/0.543	−0.004	−0.130/0.119
Autonomy	0.066	−0.188/0.418	0.108	0.024/0.299
Potential for development		0.112/0.516	0.315	0.278/0.511
Financial reward	0.094	−0.056/0.216	0.063	−0.023/0.123
Influence of work on private life	−.0.143	−0.485/0.12	0.91	0.005/0.236
R^2^ Model	R^2^ = 0.567		R^2^ = 0.412	

Model multiple regression analysis: outcome = mean job satisfaction. Predictor(s) demographic characteristics = age / total years of experience in profession / total years in current job/hours per week worked plus work-related elements of job satisfaction = personnel & organization/ work demands and tasks/, social support at work/ working relationships/ workplace agreements & referrals/autonomy/potential for development/financial reward/influence of work on private life

^a^*n* = 103

^b^*n* = 405

Our model shows that for hospital midwives, the domains ‘working hours per week’ (*p* = 0.022), ‘potential for development’ (*p* = 0.003) ‘workplace agreements and referrals’ (*p* = 0.010) and ‘total years in the profession’ (*p* = 0.034) were all significant predictors for job satisfaction.

For primary-care midwives, the domains ‘potential for development’ (*p* = 0.001), ‘social support at work’ (*p* = 0.001), ‘work demands and tasks’ (*p* = 0.001), ‘autonomy’ (*p* = 0.022) and ‘influence of work on private life’ (*p* = 0.041) were all found to be significant predictors for job satisfaction.

The R^2^ for hospital midwives was .57 and for primary care midwives it was .41.

## Discussion

Our study is the first national study to compare job satisfaction between hospital and primary-care midwives. Based on the total number of midwives practising in the Netherlands (*n* = 3150) our survey has a collective response rate of 17% of all midwives. Our study showed that all midwives in our survey were satisfied with their jobs and that there was no significant difference between hospital midwives and primary-care midwives in terms of overall satisfaction. While this is broadly in line with other, smaller-scale studies on the subject [16, 17] there are some important differences.

We found that the strongest predictor for job satisfaction for both groups of midwives was the domain ‘potential for development’. This suggests that the facilitation of ways in which midwives can develop should be of primary concern to policy-makers. This is especially important in the context of midwifery service provision and changing models of care both in the Netherlands and internationally.

Midwives’ professional organizations are becoming increasingly aware of the importance of the components of job satisfaction. Within the Netherlands the KNOV has campaigned for the reduction in the caseloads of primary-care midwives and for an increase in the potential for development of the midwives’ scope of practice. In the UK, in response to a Government white paper on healthcare reform, the Royal College of Midwives (RCM) stated that almost half of all midwives surveyed wanted further training and development [22].

Other healthcare professionals have also commented on the importance of the potential for development within their role. For example, Bjorka et al. [23] found that the introduction of career ladders significantly improved job satisfaction in Norwegian nurses. Clearly, an understanding of the importance of the potential for development to midwives can be advantageous when considering the strategic planning of maternity services.

Potential for development can also be seen in terms of the ongoing need to develop professionally, such as a commitment to life-long learning. Professional development will of course, have cost implications for employers and midwives themselves [24]. For Dutch midwives, who have different terms of remuneration according to the type and place of their practice (which, in turn, may be affected by new models of care), this is a particularly important fact for policymakers to consider [8, 25].

Insufficient opportunity for development was also associated with higher rates of burnout among Australian hospital midwives [26] . In addition, Yoshida and Sandall [27] found that rates of occupational burnout are not only higher among midwives than those in comparable professions, but also that the incidence of ‘midwife burnout’ is on the rise. Like ‘potential for development’, burnout is linked to other aspects of job satisfaction [28].

The domain ‘workplace agreements’ (which contains factor-statements relating to workplace protocols) was also a significant predictor for job satisfaction for hospital midwives. Over the last 20 years, as evidence-based practice has become more common, widespread reliance on protocols has become the norm [29]. However, it is important to note that these protocols need to be of sufficient quality and up-to-date in order to ensure that they are fit-for-purpose. A recent national survey of the proliferation of labour-ward protocols in the Netherlands found that those protocols were of variable quality and often out of date [30]. Over reliance on poor protocols may lead to a false sense of security and may harm patient care [31].

For hospital midwives in our survey, the domain ‘working hours-per-week’ was also a significant predictor of job satisfaction. Work/life balance is an oft-cited issue in the promotion of job satisfaction [32, 33]. Internationally, the introduction of flexible work schedules has been suggested as a way to mitigate work constraints [34]. Among midwives in the Netherlands it has been hypothesized that having more regular hours may be the reason for the shift to hospital practice [35]. Two out of three Dutch midwives work part-time and most new graduates indicate a desire to work part-time by the time they reach the age of 30 [36].

There is evidence that shows that older workers contribute more value in the workplace than their younger counterparts [37]. Studies of nurses indicate that keeping staff in-post, especially those with more experience is also linked to job satisfaction [38]. Our survey certainly demonstrates that for hospital midwives in the Netherlands, having more experience led to higher levels of satisfaction.

The domain ‘work demands and tasks’ was a significant predictor of job satisfaction for primary-care midwives in our survey. For midwives and other healthcare providers, the pressure of work is cited as one of the main contributing factors leading to reduced motivation, increased levels of sickness, and ultimately, to leaving the profession [39–42]. This situation is mirrored in the Netherlands where Dutch primary-care midwives receive payment according to how many clients they care for during pregnancy and birth. This payment schedule is set nationally and means that Dutch primary-care midwives must accept a high caseload in order to meet the nationally agreed salary for full-time employment. Although in recent years the norm of the number of clients in the average primary-care midwife’s caseload has been reduced (from 112 to 105 per year^3^), there are calls to reduce it even further [43]. Our survey suggests that this is a matter of importance for primary-care midwives in terms of their job satisfaction.

The domain ‘social support at work’ is also identified as a significant predictor of job satisfaction for primary-care midwives. This is important in the context of continuity-of-care models (COCM) characteristic of midwifery service provision in the Netherlands. While some studies report lower rates of burnout for primary-care midwives [44], others report higher levels of occupational stress and burnout among primary-care midwives [45]. Nevertheless, in the Netherlands where the caseload norm of primary-care midwives are high (105 per year) the pressure of work can lead to a sense of isolation, stress and burnout among midwives [42]. Research has confirmed the need to ‘care for the carer’, with reports of organizational or systematic stress, and even post-traumatic stress disorder, in healthcare professionals who feel isolated and lack good support networks at work [46]. The recent initiative by the UK RCM [47]: ‘Caring for You Charter’ is a good example of midwives’ professional organizations encouraging key stakeholders to commit to improving support networks in the workplace. A system of formal mentoring and support among Dutch midwives (particularly in the context of new models of care) is likely to contribute to increased satisfaction levels. Formal arrangements for mentoring in midwifery settings have been shown to have merit [48].

Studies consistently confirm that elements which allow for a ‘sense of control’ are important to job satisfaction [17, 46, 49]. We also found this to be true in our study. The ability to work autonomously and the influence of work on private life (being able to “leave work at work”) were significant predictors of job satisfaction for primary-care midwives in our study. This has been reported in other studies of midwives [50, 51], general practitioners [52], and nurses [53]. Primary-care midwives in the Netherlands are autonomous practitioners and they see this as an important facet of their work. Any changes to working practices of midwives should protect or improve their level of autonomy.

Primary-care midwives indicated that their level of job satisfaction was higher when their work least impinged on their private life. This is however, made difficult by the fact that primary-care midwives in the Netherlands often work long periods on-call. The European Working Time-Directive [54] dictates maximum working hours and minimal rest periods, but exempts self-employed persons. The Dutch Healthcare Inspectorate has said that self-employed primary-care midwives fall under the guidance issued for doctors [55], which states that there should be “a maximum of five on-call shifts per week”. Because 24 or even 48 h shifts are not unknown in the Netherlands [6], there is a clear need to establish what constitutes a safe maximum working period for primary-care midwives and to balance this with service needs.

### Strengths and limitations

Our survey has explored the views of Dutch midwives related to their job satisfaction. It provides a deeper understanding of the underlying differences in the factors that contribute to the job satisfaction of Dutch midwives and how those factors relate to their working practices.

Our respondents came from most areas of the Netherlands and they mirror the population of Dutch midwives. We used a validated questionnaire and piloted our survey with hospital and primary-care midwives.

Our study is limited by the fact that our sample was self-selected and was dependent on willingness to share our survey on the part of the person responsible for the email in the practice setting (primary-care or hospital). We attempted to compensate for this by placing notices encouraging all midwives to complete the survey on the midwives’ professional organization’ website. However, more primary-care midwives than hospital midwives are members of the midwives’ professional organization (94% vs 52%^2^), a factor that may have contributed to the lower proportion of hospital midwives accessing the questionnaire via the KNOV route. Nevertheless, since we received replies from 97% of all hospitals in the Netherlands, we are confident that the number and geographic distribution of responders was high enough to provide valid insights.

### Relevance

Midwifery in the Netherlands is changing; nationally, there is a trend toward hospital employment for midwives [2] and new models of care are being introduced. These changes have the potential to alter the way midwives work. A deeper understanding of what midwives themselves see as important contributors to their job satisfaction, before the introduction of substantial change, is necessary and prudent.

Our study offers a better understanding of what midwives say is important to them; its message is also relevant internationally, where it has been stated that the midwifery profession has the opportunity to learn from changes to practice in the Netherlands [56].

## Conclusion

Overall, Dutch midwives are satisfied with their jobs. Significant differences in predictors for job satisfaction between hospital and primary-care midwives in the Netherlands exist. Given job satisfaction affects the quality of care (and satisfaction with that care), these differences are important to consider when planning workforce needs and should be used by policymakers in the Netherlands and elsewhere when planning new models of care.

## Additional files

Additional file 1: English version of questionnaire. (PDF 224 kb)

Additional file 2: Nederlands (Dutch) version of questionnaire. (DOCX 23 kb)

## Data Availability

Data were stored electronically in an encrypted database. Copies of datasets used for this study may be available from the corresponding author on reasonable request.

## Abbreviations

COCM: Continuity of care model
KNOV: Koninklijke Nederlandse Organisatie van Verloskundigen (Royal Dutch Organisation of Midwives)
LQWLQ: The Leiden Quality of Work Life Questionnaire
MCAR: Missing completely at random
NVOG: Nederlandse Vereniging van Obstetrie en Gynaecologie (Dutch Society of Obstetricians and Gynaecologists)
RCM: The Royal College of Midwives of the UK
