# Supplementary material for: Are midwives in the Netherlands satisfied with their jobs? A systematic examination of satisfaction levels among hospital and primary-care midwives in the Netherlands
Source: BMC Health Serv Res. 2019 Nov 13;19:832. doi: 10.1186/s12913-019-4454-x (PMC6854733; doi:10.1186/s12913-019-4454-x)
Supplement: Supplementary file 1 — English version of questionnaire. (PDF 224 kb) [file 12913_2019_4454_MOESM1_ESM.pdf]

Incas 2 Questionnaire

Inleiding

Inleiding

Deze vragenlijst heeft tot doel de huidige tevredenheid onder zorgverleners betrokken bij zwangerschapszorg te kwantificeren.

Deze vragenlijst wordt gebruikt voor zowel de INCAS studie (onderzoek naar integrale zorg door Midwifery Science van het VUmc) als de CONNECT-IN studie (studie naar CenteringPregnancy door TNO en LUMC).Om u niet teveel te belasten is in overleg besloten deze vragenlijst voor beide studies te combineren

Dit vragenlijstonderzoek zal bij de evaluatie van de integrale zorgpilot herhaald worden.
Voor deelnemers aan de CONNECT-IN studie is aan het einde nog een aantal vragen toegevoegd over de ervaringen met CenteringPregnancy.

Wij vragen u vriendelijk om uw medewerking aan dit onderzoek te verlenen door het invullen van deze vragenlijst. Het invullen zal ongeveer twintig minuten in beslag nemen.

Mocht u vragen hebben over de INCAS studie dan kunt u contact opnemen met dr. Corine Verhoeven, c.verhoeven@vumc.nl, tel. 020-4448406 of 06-51853746.

Hebt u vragen over de CONNECT-IN studie dan kunt u contact opnemen met Birgit Bruinsma MSc, birgit.bruinsma@tno.nl, tel 071-5268605.

Bij voorbaat hartelijk dank,

Het INCAS-2 onderzoeksteam.
Het CONNECT-IN onderzoeksteam.

Waar in deze vragenlijst ‘zij’ of ‘haar’ staat, wordt ook ‘hij’ of ‘hem’ bedoeld.

In de vragenlijst wordt 'cliënten' gebruikt voor cliënten/patiënten.

In deze vragenlijst gebruiken we 'keten' voor alle zorgverleners in de geboortezorg in de regio waar u mee samenwerkt.

In de vragenlijst wordt met 'organisatie' de instelling/praktijk waar u werkzaam bent bedoeld.

In de vragenlijst bedoelen we met 'collega’s' alle personeel werkzaam binnen uw organisatie.

In de vragenlijst wordt met ‘protocol’ alle protocollen, werkafspraken, richtlijnen en/of werkprocedures bedoeld.

Algemene vragen

***1. Behoort het uitoefenen van de verloskundige of obstetrische zorg tot uw functie?**

- ◯ Ja
- ◯ Nee

***2. Wat is uw geslacht?**

- [ ] Man
- [ ] Vrouw

***3. Binnen de verloskundige zorg ben ik werkzaam als:**

- [ ] Klinische verloskundige
- [ ] Eerstelijns verloskundige
- [ ] gynaecoloog
- [ ] O & G verpleegkundig
- [ ] Niet O & G Verpleegkundige
- [ ] Kraamverzorgende
- [ ] Anders namelijk

***4. Hoeveel jaar werkervaring hebt u binnen uw beroep in de geboortezorg? (rond af in hele jaren)**

jaren ervaring

***5. Hoe bent u werkzaam?**

- ○ In loondienst
- ○ Zelfstandig ondernemer
- ○ Waarnemer
- ○ Anders namelijk

***6. Wat is uw leeftijd?**

leeftijd in jaren

***7. Hoeveel uur werkt u gemiddeld per week?**

Uur per week

Personeel en organisatie

***8. Binnen mijn organisatie is er voldoende personeel aanwezig om goede zorg te kunnen bieden.**

- ◯ helemaal mee oneens
- ◯ mee oneens
- ◯ mee eens
- ◯ helemaal mee eens

***9. Bij afwezigheden (bijv. door ziekte) wordt in mijn organisatie voldoende vervangend personeel ingezet.**

- ◯ helemaal mee oneens
- ◯ mee oneens
- ◯ mee eens
- ◯ helemaal mee eens

***10. In mijn organisatie is er voldoende personeel met ervaring.**

- ◯ helemaal mee oneens
- ◯ mee oneens
- ◯ mee eens
- ◯ helemaal mee eens

***11. In mijn keten is de eerstelijns verloskundige zorg in het algemeen goed georganiseerd.**

- ◯ helemaal mee oneens
- ◯ mee oneens
- ◯ mee eens
- ◯ helemaal mee eens

***12. In mijn keten is de tweedelijns verloskundige zorg in het algemeen goed georganiseerd.**

- ◯ helemaal mee oneens
- ◯ mee oneens
- ◯ mee eens
- ◯ helemaal mee eens

***13. In mijn keten is de derdelijns verloskundige zorg in het algemeen goed georganiseerd.**

- ◯ helemaal mee oneens
- ◯ mee oneens
- ◯ mee eens
- ◯ helemaal mee eens

***14. In mijn keten is de kraamzorg in het algemeen goed georganiseerd.**

- ◯ helemaal mee oneens
- ◯ mee oneens
- ◯ mee eens
- ◯ helemaal mee eens

Werkeisen en taken

***15. Ik heb voldoende tijd om goede zorg te verlenen aan cliënten.**

- helemaal mee oneens
- mee oneens
- mee eens
- helemaal mee eens

***16. Ik heb voldoende tijd om mijn vakliteratuur bij te houden.**

- helemaal mee oneens
- mee oneens
- mee eens
- helemaal mee eens

***17. Er zijn regelmatig te veel cliënten aan mijn zorgen toevertrouwd.**

- helemaal mee oneens
- mee oneens
- mee eens
- helemaal mee eens

***18. Ik kom regelmatig niet toe aan mijn cliënt gebonden taken.**

- helemaal mee oneens
- mee oneens
- mee eens
- helemaal mee eens

***19. Ik functioneer regelmatig op de grenzen van mijn (fysieke) mogelijkheden.**

- helemaal mee oneens
- mee oneens
- mee eens
- helemaal mee eens

***20. Ik heb te veel verschillende taken.**

- helemaal mee oneens
- mee oneens
- mee eens
- helemaal mee eens

Sociale steun op werkgebied en saamhorigheid

***21. Mijn direct leidinggevende heeft aandacht voor persoonlijke omstandigheden van haar medewerkers.**

○ helemaal mee oneens ○ mee oneens ○ mee eens ○ helemaal mee eens ○ nvt

***22. Ik kan rekenen op de steun van mijn direct leidinggevende als ik een probleem heb in mijn werk.**

○ helemaal mee oneens ○ mee oneens ○ mee eens ○ helemaal mee eens ○ nvt

***23. Ik heb vertrouwen in mijn direct leidinggevende.**

○ helemaal mee oneens ○ mee oneens ○ mee eens ○ helemaal mee eens ○ nvt

***24. Mijn direct leidinggevende verdedigt mijn belangen naar anderen toe.**

○ helemaal mee oneens ○ mee oneens ○ mee eens ○ helemaal mee eens ○ nvt

***25. Ik voel mij door mijn direct leidinggevende gewaardeerd.**

○ helemaal mee oneens ○ mee oneens ○ mee eens ○ helemaal mee eens ○ nvt

***26. Ik krijg feedback van mijn leidinggevende over mijn functioneren.**

○ helemaal mee oneens ○ mee oneens ○ mee eens ○ helemaal mee eens ○ nvt

Samenwerking

***27. De onderlinge samenwerking binnen mijn organisatie is goed.**

○ helemaal mee oneens ○ mee oneens ○ mee eens ○ helemaal mee eens

***28. De onderlinge samenwerking met mijn ketenpartners is goed.**

○ helemaal mee oneens ○ mee oneens ○ mee eens ○ helemaal mee eens

***29. Andere zorgverleners binnen mijn beroepsgroep ervaar ik meer als collega's dan als concurrenten.**

○ helemaal mee oneens ○ mee oneens ○ mee eens ○ helemaal mee eens

***30. De andere ketenpartners ervaar ik meer als collega's dan als concurrenten.**

○ helemaal mee oneens ○ mee oneens ○ mee eens ○ helemaal mee eens

***31. Binnen mijn organisatie verloopt de onderlinge communicatie goed.**

○ helemaal mee oneens ○ mee oneens ○ mee eens ○ helemaal mee eens

***32. Met mijn ketenpartners verloopt de communicatie goed.**

○ helemaal mee oneens ○ mee oneens ○ mee eens ○ helemaal mee eens

***33. Binnen mijn organisatie heb ik vertrouwen in de bekwaamheid van mijn collega's.**

○ helemaal mee oneens ○ mee oneens ○ mee eens ○ helemaal mee eens

***34. Ik heb vertrouwen in de bekwaamheid van mijn ketenpartners.**

○ helemaal mee oneens ○ mee oneens ○ mee eens ○ helemaal mee eens

***35. Binnen mijn organisatie voel ik me door mijn collega's gewaardeerd.**

○ helemaal mee oneens ○ mee oneens ○ mee eens ○ helemaal mee eens

***36. Ik voel me door mijn ketenpartners gewaardeerd.**

○ helemaal mee oneens ○ mee oneens ○ mee eens ○ helemaal mee eens

***37. In mijn organisatie bekritiseren medewerkers elkaar op een hinderlijke manier.**

○ helemaal mee oneens ○ mee oneens ○ mee eens ○ helemaal mee eens

***38. Mijn ketenpartners bekritiseren elkaar op een hinderlijke manier.**

○ helemaal mee oneens ○ mee oneens ○ mee eens ○ helemaal mee eens

***39. Binnen mijn organisatie bieden mijn collega's een helpende hand als dat nodig is.**

○ helemaal mee oneens ○ mee oneens ○ mee eens ○ helemaal mee eens

***40. Mijn ketenpartners bieden mij een helpende hand als dat nodig is.**

- ◯ helemaal mee oneens
- ◯ mee oneens
- ◯ mee eens
- ◯ helemaal mee eens

***41. Binnen mijn organisatie geven mijn collega's mij emotionele steun als ik het moeilijk heb.**

- ◯ helemaal mee oneens
- ◯ mee oneens
- ◯ mee eens
- ◯ helemaal mee eens

***42. Mijn ketenpartners geven mij emotionele steun als ik het moeilijk heb.**

- ◯ helemaal mee oneens
- ◯ mee oneens
- ◯ mee eens
- ◯ helemaal mee eens

***43. Binnen de keten vind ik de volgende zorgverleners goed bereikbaar:**

	eerstelijns verloskundigen	kraamzorg	gynaecologen	tweedelijns verloskundigen	verpleegkundigen	praktijk ondersteuners
helemaal mee oneens	◯	◯	◯	◯	◯	◯
mee oneens	◯	◯	◯	◯	◯	◯
mee eens	◯	◯	◯	◯	◯	◯
helemaal mee eens	◯	◯	◯	◯	◯	◯

anders namelijk

[ ]

***44. Ik heb vertrouwen in een goede samenwerking binnen de toekomstige verloskundige organisatie.**

- ◯ helemaal mee oneens
- ◯ mee oneens
- ◯ mee eens
- ◯ helemaal mee eens

Afspraken en overdracht

***45. Ik krijg bij overdracht van een cliënt meestal volledige en juiste informatie overgedragen.**

- ◯ helemaal mee oneens
- ◯ mee oneens
- ◯ mee eens
- ◯ helemaal mee eens

***46. Binnen mijn organisatie zijn protocollen vaak onduidelijk.**

- ◯ helemaal mee oneens
- ◯ mee oneens
- ◯ mee eens
- ◯ helemaal mee eens

***47. Binnen mijn organisatie ontbreken protocollen vaak.**

- ◯ helemaal mee oneens
- ◯ mee oneens
- ◯ mee eens
- ◯ helemaal mee eens

***48. Binnen mijn organisatie zijn protocollen in de praktijk vaak niet uitvoerbaar.**

- ◯ helemaal mee oneens
- ◯ mee oneens
- ◯ mee eens
- ◯ helemaal mee eens

***49. Protocollen die gemaakt zijn binnen onze beroepsgroep worden over het algemeen goed nageleefd / gevolgd.**

- ◯ helemaal mee oneens
- ◯ mee oneens
- ◯ mee eens
- ◯ helemaal mee eens

Autonomie

***50. Ik moet voortdurend uitvoeren wat anderen mij opdragen.**

- ◯ helemaal mee oneens
- ◯ mee oneens
- ◯ mee eens
- ◯ helemaal mee eens

***51. Mijn werk biedt me de ruimte om zelf beslissingen te nemen.**

- ◯ helemaal mee oneens
- ◯ mee oneens
- ◯ mee eens
- ◯ helemaal mee eens

***52. Ik heb inspraak in beslissingen die mijn werk aangaan.**

- ◯ helemaal mee oneens
- ◯ mee oneens
- ◯ niet mee eens, niet mee oneens
- ◯ mee eens
- ◯ helemaal mee eens

***53. Ik kan zelf bepalen wanneer ik cliëntgebonden en niet-cliëntgebonden taken doe.**

- ◯ helemaal mee oneens
- ◯ mee oneens
- ◯ mee eens
- ◯ helemaal mee eens

***54. Ik kan mijn werk naar eigen inzicht uitvoeren.**

- ◯ helemaal mee oneens
- ◯ mee oneens
- ◯ mee eens
- ◯ helemaal mee eens

***55. Ik verwacht in de toekomstige verloskundige organisatie professionele autonomie te verliezen.**

- ◯ helemaal mee oneens
- ◯ mee oneens
- ◯ mee eens
- ◯ helemaal mee eens
- ◯ nvt

Ontwikkelingsmogelijkheden

***56. Ik heb veel routinematige taken.**

○ helemaal mee oneens ○ mee oneens ○ mee eens ○ helemaal mee eens

***57. Mijn werk is afwisselend.**

○ helemaal mee oneens ○ mee oneens ○ mee eens ○ helemaal mee eens

***58. Mijn werk biedt mij de mogelijkheid nieuwe dingen te leren.**

○ helemaal mee oneens ○ mee oneens ○ mee eens ○ helemaal mee eens

***59. In mijn werk kan ik mij voldoende ontplooien.**

○ helemaal mee oneens ○ mee oneens ○ mee eens ○ helemaal mee eens

***60. In mijn werk heb ik de mogelijkheid om mijn capaciteiten verder te ontwikkelen.**

○ helemaal mee oneens ○ mee oneens ○ mee eens ○ helemaal mee eens

Financiële waardering en arbeidstevredenheid

***61. Ik word goed betaald voor het werk dat ik doe.**

- ◯ helemaal mee oneens
- ◯ mee oneens
- ◯ mee eens
- ◯ helemaal mee eens

***62. Ik verwacht dat ik in de toekomst goed betaald word voor het werk dat ik doe.**

- ◯ helemaal mee oneens
- ◯ mee oneens
- ◯ mee eens
- ◯ helemaal mee eens

***63. Ik ben tevreden met mijn baan.**

- ◯ helemaal mee oneens
- ◯ mee oneens
- ◯ mee eens
- ◯ helemaal mee eens

Invloed werk op de thuissituatie

***64. Mijn werk kan ik thuis moeilijk van mij afzetten.**

- ◯ helemaal mee oneens
- ◯ mee oneens
- ◯ mee eens
- ◯ helemaal mee eens

***65. Door mijn werk kan ik verplichtingen thuis vaak niet nakomen.**

- ◯ helemaal mee oneens
- ◯ mee oneens
- ◯ mee eens
- ◯ helemaal mee eens

***66. Door mijn werk kom ik onvoldoende toe aan ontspannende bezigheden.**

- ◯ helemaal mee oneens
- ◯ mee oneens
- ◯ mee eens
- ◯ helemaal mee eens

***67. Doet uw organisatie mee aan de CONNECT-IN studie (naar het effect van CenteringPregnancy)?**

- ◯ Ja
- ◯ Nee

Aanvullende vragen ALLEEN voor ConnectIN studie (praktijken die mee doen a...

Individuele prenatale zorg

***68. Behoort het uitvoeren van prenatale controles tot uw werkzaamheden?**

- ◯ Ja: ga door met rest van vragenlijst
- ◯ Nee: einde vragenlijst

***69. Hoe beoordeelt u de kwaliteit van de individuele prenatale zorg die uw organisatie aanbiedt? Geef een rapportcijfer van 0 tot 10 (waarbij 0 de slechtst mogelijke en 10 de best mogelijke prenatale zorg is)**

○ 1 ○ 2 ○ 3 ○ 4 ○ 5 ○ 6 ○ 7 ○ 8 ○ 9 ○ 10

***70. Hoe tevreden bent u over hoe ú zelf de individuele prenatale zorg geeft?**

○ heel erg tevreden ○ erg tevreden ○ een beetje tevreden ○ ontevreden

***71. Hoe beoordeelt u de relatie met uw cliënten/patiënten binnen de individuele prenatale zorg? Geef een rapportcijfer van 0 tot 10 (waarbij 0 de slechtst mogelijke en 10 de best mogelijke prenatale zorg is)**

○ 1 ○ 2 ○ 3 ○ 4 ○ 5 ○ 6 ○ 7 ○ 8 ○ 9 ○ 10

Feitelijke tijdsbesteding prenatale zorg

***72. Hoeveel cliënten ziet u gemiddeld per uur tijdens een regulier prenataal spreekuur?**

aantal clienten

***73. Hoeveel minuten direct contact hebt u gemiddeld met een cliënt tijdens een regulier prenataal consult?**

minuten contact-tijd

***74. Hoe beoordeelt u deze tijdsduur waarin u direct contact hebt met de zwangere cliënt tijdens individuele prenatale controles?**

- ◯ ruim voldoende
- ◯ voldoende
- ◯ onvoldoende
- ◯ ruim onvoldoende

***75. Hoeveel tijd (in minuten) besteedt u gemiddeld aan de voorbereiding van een regulier prenataal spreekuur?**

minuten voorbereiding

***76. Hoeveel tijd (in minuten) besteedt u gemiddeld aan de afwerking van een regulier prenataal spreekuur? (Bijvoorbeeld rapportage, bloedresultaten, brieven schrijven, echo-uitslagen)**

minuten afronden

***77. Hoe beoordeelt u de tijd die u investeert in het geheel van voorbereiding, uitvoer en afwerking van de individuele prenatale controles?**

- ◯ kost helemaal niet veel tijd
- ◯ kost niet veel tijd
- ◯ kost redelijk veel tijd
- ◯ kost erg veel tijd

Verwachtingen ten aanzien van CP

De volgende stellingen gaan over uw ervaringen met CP (als u al gestart bent) of verwachtingen over (als u nog met starten) de implementatie en uitvoer van CenteringPregnancy in de praktijk. U kunt bij iedere stelling aangeven in hoeverre u het ermee eens bent.

***78. Ik vind het belangrijk om door middel van CenteringPregnancy betere zwangerschapsuitkomsten bij mijn cliënten te bereiken.**

- ◯ helemaal mee oneens
- ◯ mee oneens
- ◯ mee eens
- ◯ helemaal mee eens

***79. Ik verwacht dat door het volgen van CenteringPregnancy daadwerkelijk betere zwangerschapsuitkomsten bij mijn cliënten worden bereikt.**

- ◯ zeer zeker niet
- ◯ zeker niet
- ◯ zeker wel
- ◯ zeer zeker wel

***80. Ik vind het tot mijn taak als verloskundige horen om cliënten te motiveren om deel te nemen aan CenteringPregnancy.**

- ◯ helemaal mee oneens
- ◯ mee oneens
- ◯ mee eens
- ◯ helemaal mee eens

***81. Cliënten zijn over het algemeen tevredener als ook CenteringPregnancy wordt aangeboden.**

- ◯ helemaal mee oneens
- ◯ mee oneens
- ◯ mee eens
- ◯ helemaal mee eens

***82. Cliënten stemmen over het algemeen toe in deelname aan CenteringPregnancy.**

- ◯ helemaal mee oneens
- ◯ mee oneens
- ◯ mee eens
- ◯ helemaal mee eens

***83. Deelneemsters aan CenteringPregnancy zijn aanwezig bij vrijwel alle sessies.**

- ◯ helemaal mee oneens
- ◯ mee oneens
- ◯ mee eens
- ◯ helemaal mee eens

***84. Deelneemsters aan CenteringPregnancy volgen over het algemeen deze vorm van zorg en gaan niet terug naar de individuele zorg.**

- ◯ helemaal mee oneens
- ◯ mee oneens
- ◯ mee eens
- ◯ helemaal mee eens

***85. Partners van cliënten willen over het algemeen aanwezig zijn bij de CenteringPregnancy sessies.**

- ◯ helemaal mee oneens
- ◯ mee oneens
- ◯ mee eens
- ◯ helemaal mee eens

***86. Ik kan op voldoende hulp van mijn collega's rekenen bij het implementeren van CenteringPregnancy (planning, voorbereiding, volgen training).**

- ◯ helemaal mee oneens
- ◯ mee oneens
- ◯ mee eens
- ◯ helemaal mee eens

***87. Ik kan op voldoende hulp van mijn collega's rekenen bij het uitvoeren van CenteringPregnancy.**

◯ helemaal mee oneens ◯ mee oneens ◯ mee eens ◯ helemaal mee eens

***88. Het lukt mij om voldoende zwangeren enthousiast te maken voor CenteringPregnancy om iedere 4 tot 6 weken een nieuwe groep te starten.**

◯ zeer zeker niet ◯ zeker niet ◯ zeker wel ◯ zeer zeker wel

***89. Het lukt mij om een CenteringPregnancy-groep te begeleiden.**

◯ zeer zeker niet ◯ zeker niet ◯ zeker wel ◯ zeer zeker wel

Dit is het einde van de vragenlijst.

Namens de onderzoek teams van CONNECT-IN en INCAS 2 studies, hartelijk dank voor uw deelname.
