# Supplementary material for: Are midwives in the Netherlands satisfied with their jobs? A systematic examination of satisfaction levels among hospital and primary-care midwives in the Netherlands
Source: BMC Health Serv Res. 2019 Nov 13;19:832. doi: 10.1186/s12913-019-4454-x (PMC6854733; doi:10.1186/s12913-019-4454-x)
Supplement: Supplementary file 2 — Nederlands (Dutch) version of questionnaire. (DOCX 23 kb) [file 12913_2019_4454_MOESM2_ESM.docx]

***Incas 2 Questionnaire***

**Introduction**

Introduction: The goal of this questionnaire is to quantify the current levels of satisfaction among caregivers involved in maternity care.

This questionnaire will be used for the INCAS study (research into integrated models of care, undertaken by the department of Midwifery Science at the VuMc) and by the CONNECT-IN study (study into Centering Pregnancy undertaken by TNO and LUMC). Following discussion and in order not to overtax respondents it was decided to combine this questionnaire for both studies.

This research questionnaire will be repeated during evaluation of the integrated-care pilots.

For participants of the CONNECT-IN study only, there are a number of questions at the end of the questionnaire related to your experiences with Centering Pregnancy.

We ask for your friendly cooperation with this research by completing this questionnaire. It should take approximately twenty minutes of your time.

If you have questions regarding the INCAS study then you can contact Dr. Corine Verhoeven at [c.verhoeven@vumc.nl](mailto:c.verhoeven@vumc.nl) tel. 020-4448406

If you have questions regarding to the CONNECT-IN study then you can contact Birgit Bruinsma MSc at [birgit.bruinsma@tno.nl](mailto:birgit.bruinsma@tno.nl) tel. 071-5268605

Thank you in advance,

The INCAS research team.

The CONNECT-IN research team.

Where in this questionnaire we use ‘she’ or ‘her’, we mean also ‘he’ or ‘him’.

In this questionnaire we use ‘clients’ to mean also patients.

In this questionnaire we use ‘multi-disciplinary team’ to mean all caregivers involved in maternity care in the healthcare region where you work.

In this questionnaire we use ‘organization’ to mean the facility or practice where you work.

In this questionnaire we use ‘colleague’ to mean all personnel working within your organization.

In this questionnaire we use ‘protocol’ to mean all protocols, work agreements, guidance-documents and/or work procedures.

***Incas 2 Questionnaire***

**General questions**

Where relevant to your function all questions marked with * are compulsory

1* In your function are you involved with midwifery or obstetric care?

Yes No

If *no* please stop questionnaire

***Incas 2 Questionnaire***

2* What is your gender?

Man Woman

3* Within midwifery care I am employed as:

Clinical midwife

Primary-care midwife

Gynaecologist

Obstetric Nurse

General Nurse

Care assistant

Other, please specify:

4* Within maternity care how many years of experience do you have in your profession? (round-off to complete years)

Years of experience:

5* How long have you worked in your current job? (round-off to complete years)

Years in current job:

6* How are you employed?

Salaried

Self-employed

Temp

Other, please specify:

7* What is your age?

Age in years:

8* How many hours on average do you work per week?

Hours per week:

***Incas 2 Questionnaire***

**Domain: Personnel and organization**

9* Within my organization there are enough staff available to offer good care

Completely disagree disagree agree completely agree

10* Within my organization in the event of absence (for example through sickness) there are enough replacement staff brought in

Completely disagree disagree agree completely agree

11* Within my organization there are enough experienced staff

Completely disagree disagree agree completely agree

12* Within the multi-disciplinary team primary-care midwifery is well organized

Completely disagree disagree agree completely agree

13* Within the multi-disciplinary team hospital midwifery care is well organized

Completely disagree disagree agree completely agree

14* Within the multi-disciplinary team tertiary (University hospital) midwifery care is well organized

Completely disagree disagree agree completely agree

15* Within the multi-disciplinary team the home-help (by care assistants) is well organized

Completely disagree disagree agree completely agree

16* I expect that there will be enough staff in the midwifery organization of the future

Completely disagree disagree agree completely agree

***Incas 2 Questionnaire***

**Work demands and tasks**

17* I have enough time to give good care to my clients

Completely disagree disagree agree completely agree

18* I have enough time to keep-up to-date with my professional journals

Completely disagree disagree agree completely agree

19* I regularly have too many clients in my care

Completely disagree disagree agree completely agree

20* I am regularly unable to get to client-related tasks

Completely disagree disagree agree completely agree

21* I regularly have to function at the limit of my physical capabilities

Completely disagree disagree agree completely agree

22* I have too many differing tasks

Completely disagree disagree agree completely agree

23* I expect to have enough time to do my work well in the midwifery organization of the future

Completely disagree disagree agree completely agree

***Incas 2 Questionnaire***

**Social support and cohesion in the workplace**

24* My direct supervisor pays enough attention to the personal circumstances of her employees

Completely disagree disagree agree completely agree

25* I can expect the support of my direct supervisor if I experience problems in my work

Completely disagree disagree agree completely agree

26* I trust my direct supervisor

Completely disagree disagree agree completely agree

27* My direct supervisor defends my importance to others

Completely disagree disagree agree completely agree

28* I feel valued by my direct supervisor

Completely disagree disagree agree completely agree

29* I get feedback regarding my performance from my direct supervisor

Completely disagree disagree agree completely agree

30* I expect that social support at work will be good in the midwifery organization of the future

Completely disagree disagree agree completely agree

***Incas 2 Questionnaire***

**Working relationships**

31* Between staff working within my organization working relationships are good

Completely disagree disagree agree completely agree

32* Between staff working within the multidisciplinary team working relationships are good

Completely disagree disagree agree completely agree

33* I regard other caregivers from within my profession more as colleagues than competitors

Completely disagree disagree agree completely agree

34* I regard other members of the multidisciplinary team more as colleagues than competitors

Completely disagree disagree agree completely agree

35* Between staff working within my organization communication is good

Completely disagree disagree agree completely agree

36* Between staff working within the multidisciplinary team communication is good

Completely disagree disagree agree completely agree

37* Within my organization I trust the abilities of my colleagues

Completely disagree disagree agree completely agree

38* Within the multidisciplinary team I trust the abilities of my colleagues

Completely disagree disagree agree completely agree

39* Within my organization I feel valued by my colleagues

Completely disagree disagree agree completely agree

40* Within the multidisciplinary team I feel valued by my colleagues

Completely disagree disagree agree completely agree

41* Within my organization colleagues criticize each other in an annoying manner

Completely disagree disagree agree completely agree

42* Within the multidisciplinary team colleagues criticize each other in an annoying manner

Completely disagree disagree agree completely agree

43* Within my organization colleagues offer a helping hand if its necessary

Completely disagree disagree agree completely agree

***Incas 2 Questionnaire***

44* Within the multidisciplinary team colleagues offer a helping hand if its necessary

Completely disagree disagree agree completely agree

45* Within my organization colleagues offer emotional support if I’m having a difficult time

Completely disagree disagree agree completely agree

46* Within the multidisciplinary team colleagues offer emotional support if I’m having a difficult time

Completely disagree disagree agree completely agree

47* Within the multidisciplinary team I find the following caregivers easily accessible:

Clinical midwife: Completely disagree disagree agree completely agree

Care assistant: Completely disagree disagree agree completely agree

Gynaecologist: Completely disagree disagree agree completely agree

Hospital midwife: Completely disagree disagree agree completely agree

Nurse: Completely disagree disagree agree completely agree

Practice assistants Completely disagree disagree agree completely agree

48* I trust there will be good working relationships in the midwifery organization of the future.

Completely disagree disagree agree completely agree

***Incas 2 Questionnaire***

**Workplace agreements and handover**

49* With the handover of clients I usually get correct and complete information

Completely disagree disagree agree completely agree

50* Within my organization protocols are often unclear

Completely disagree disagree agree completely agree

51* Within my organization there is often a lack of protocols

Completely disagree disagree agree completely agree

52* Within my organization protocols are often unfeasible

Completely disagree disagree agree completely agree

53* Within my profession protocols are in general well respected/followed

Completely disagree disagree agree completely agree

54* In general, I expect that protocols will be well respected in the midwifery organization of the future

Completely disagree disagree agree completely agree

***Incas 2 Questionnaire***

**Autonomy**

55* I have to continually comply with the work I am assigned

Completely disagree disagree agree completely agree

56* My work offers me the room to make decisions for myself

Completely disagree disagree agree completely agree

57* I have a say in the decisions that affect my work

Completely disagree disagree agree completely agree

58* I can decide for myself when to complete client-related and non client-related tasks

Completely disagree disagree agree completely agree

59* I can use my own insight to carry-out my work

Completely disagree disagree agree completely agree

60* I expect to lose autonomy in the midwifery organization of the future

Completely disagree disagree agree completely agree

***Incas 2 Questionnaire***

**Potential for development**

61* I have many routine tasks

Completely disagree disagree agree completely agree

62* My work is varied

Completely disagree disagree agree completely agree

63* My work offers me the chance to learn new things

Completely disagree disagree agree completely agree

64* My work offers me the chance to develop myself

Completely disagree disagree agree completely agree

65* My work offers me the chance to further my career

Completely disagree disagree agree completely agree

66* I expect that I will be able to continue to develop my career in the midwifery organization of the future

***Incas 2 Questionnaire***

**Financial reward and job satisfaction**

67* I am well paid for the work that I do

Completely disagree disagree agree completely agree

68* I expect in the future to be well paid for the work that I do

Completely disagree disagree agree completely agree

69* If I had to choose, I’d choose this job again

Completely disagree disagree agree completely agree

70* I would like to change my job

Completely disagree disagree agree completely agree

71* I am satisfied with my job

Completely disagree disagree agree completely agree

72* I would recommend this job to a friend

Completely disagree disagree agree completely agree

73* When I applied this was the job that I wanted

Completely disagree disagree agree completely agree

74* I often have to do work that I’d rather not do

Completely disagree disagree agree completely agree

***Incas 2 Questionnaire***

**Influence of work on home life**

75* At home I find it difficult to leave my work behind me

Completely disagree disagree agree completely agree

76* Due to my workload I am often unable to fulfill my commitments at home

Completely disagree disagree agree completely agree

77* Due to my workload I have insufficient time to enjoy relaxing activities

Completely disagree disagree agree completely agree

Questions 78-100 are related to the CONNECT-IN study and were not included in the analysis and are therefore not translated.
